# Stability of BRAF V600E mutation in metastatic melanoma: new insights for therapeutic success?

**DOI:** 10.1038/bjc.2011.239

**Published:** 2011-06-21

**Authors:** L Sigalotti, E Fratta, G Parisi, S Coral, M Maio

**Affiliations:** 1 Cancer Bioimmunotherapy Unit, Department of Medical Oncology, Centro di Riferimento Oncologico, Istituto di Ricovero e Cura a Carattere Scientifico, 33081 Aviano, Italy; 2 Medical Oncology and Immunotherapy, Department of Oncology, University Hospital of Siena, Istituto Toscano Tumori, Strada delle Scotte 14, 53100 Siena, Italy

**Sir,**

We read with great interest the paper by Lin *et al* (2011) reporting a marked intratumor heterogeneity for activating BRAF mutations in primary melanomas and their positive selection along with disease recurrence or metastatic evolution. On the basis of these observations, the authors agreeably conclude that, rather than being a founder event, BRAF mutations more likely represent one of the many genetic alterations that are selected during disease progression.

The fine characterization of the ‘evolutionary biology’ of activating BRAF mutation in melanoma progression is clearly relevant as it correlates with a more aggressive course of disease (Long *et al*, 2011); nevertheless, it also bears important practical implications, as BRAF mutation in metastatic cutaneous melanoma is the molecular hallmark to select patients for treatment with the highly effective BRAF kinase inhibitors under active clinical development (Flaherty *et al*, 2010).

Prompted by the findings of Lin *et al* (2011), we asked the question whether, once melanoma has reached the metastatic stage, the BRAF mutational status undergoes additional evolution with disease progression over time and metastatic sites. To address this issue, we investigated the activating BRAF^V600E^ mutation in primary cell cultures generated from initial metastatic lesions surgically removed from 15 cutaneous melanoma patients, and in 19 subsequent metastases (Table 1). It is noteworthy that metachronous melanoma metastases, even when removed from different body parts of individual patients at a median time interval of 305 days (range 26–1318), ‘stabilized’ for the BRAF^V600E^ mutated status. The original heterozygous BRAF^V600E^ status was consistently retained in 10 out of 15 patients investigated, whereas 2 out of 15 acquired a homozygous mutated status as compared with an initial heterozygous condition (Table 1). Additionally, no BRAF^V600E^ mutation was acquired in three patients with an initial homozygous wild-type BRAF genotype (Table 1). These findings suggest that, once melanoma reaches the metastatic stage, its BRAF^V600E^ mutational status remains substantially unchanged in subsequent melanoma metastases, regardless of the time intervals to develop new metastatic lesions and site(s) of further metastatization. The lack of further modifications in the BRAF mutational condition, once melanoma has metastasized, can possibly result from absent/limited intratumor heterogeneity for the mutation in the metastatic disease. Supporting this idea, single-cell clones generated from the short-term metastatic melanoma cultures Mel 195 and Mel 313 retained the heterozygous BRAF^V600E^ mutated and homozygous wild-type genotypes of the parental metastatic cells, respectively (Figure 1).

On the basis of Lin *et al* (2011) and our data, it is likely that activating BRAF mutations are positively selected during melanoma progression until reaching the metastatic stage when the BRAF mutational status stabilizes. This finding, together with the likely limited intratumor heterogeneity of BRAF mutations, suggests that the metastatic stage of BRAF-mutated melanomas represents the most appropriate therapeutic setting for BRAF inhibitors. The highly stable BRAF status identified among metachronous melanoma metastases also bears important practical implications. In fact, any metastatic accessible tissue, either fresh or archival, regardless of the timing of metastasis and site of melanoma progression, being representative of the final BRAF mutational status of disease in a given individual, could be safely utilized to identify patients who are candidate to BRAF inhibitors. Molecular mechanism(s) of melanoma resistance to BRAF inhibitors are being initially elucidated and seem to be independent from the outgrowth of BRAF wild-type melanoma lesions (Solit and Rosen, 2011). Being the BRAF mutational status stable over time in metastatic melanoma, it is reasonable to speculate that overcoming the BRAF inhibitors-induced intrinsic resistance of melanoma cells, could allow to continue or re-challenge patients with these highly promising therapeutic agents.

## Figures and Tables

**Figure 1:**
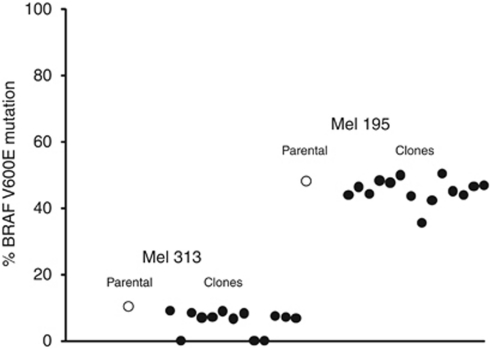
Pyrosequencing analysis of BRAF^V600E^ mutation in single-cell clones generated from Mel 195 and Mel 313 melanoma cells. Primary cultures of melanoma cells have been generated from metastatic melanoma lesions surgically removed from Mel 195 and Mel 313 patients. Single-cell clones were obtained by diluting Mel 195 and Mel 313 melanoma cell populations to three cells per ml and plating 100 *μ*l of these cell suspensions in each well of 96-well plates. Percentage of BRAF^V600E^-mutant alleles was determined in Mel 195 and Mel 313 parental melanoma cell populations, and in their derived single-cell clones, by pyrosequencing assay as described by Venesio *et al* (2008), with minor modifications.

**Table 1:** BRAF^V600E^ mutation genotyping of metachronous metastatic melanoma lesions

**Patient**	**Metastasis** a	**Site of metastasis**	**Days** b	**Percentage of mutant alleles** c	**BRAF** **V600E**^d^
Mel 90	I	M	0	57.5	WT/MT
	II	M	71	58.9	WT/MT
Mel 91	I	LN	0	55.5	WT/MT
	II	LN	238	58.8	WT/MT
Mel 120	I	LN	0	55.7	WT/MT
	III	LN	672	64.2	WT/MT
Mel 140	I	SC	0	61.4	WT/MT
	II	SC	411	70.4	WT/MT
	III	SC	728	92.9	**MT/MT**
Mel 146	I	SC	0	66.3	WT/MT
	II	LN	254	67.7	WT/MT
	III	SC	554	66.7	WT/MT
Mel 195	I	LN	0	54.6	WT/MT
	II	SC	91	46.8	WT/MT
Mel 201	I	LN	0	57.4	WT/MT
	II	P	1318	84.8	**MT/MT**
Mel 255	I	LN	0	58.8	WT/MT
	II	SC	292	52.1	WT/MT
	III	SC	299	55	WT/MT
Mel 261	I	SC	0	42.4	WT/MT
	II	SC	305	35.9	WT/MT
	III	SC	721	33.6	WT/MT
Mel 435	I	LN	0	7.2	*WT/WT*
	II	SC	139	7	*WT/WT*
Mel 458	I	SC	0	0	*WT/WT*
	II	SC	26	0	*WT/WT*
Mel 532	I	SC	0	58.2	WT/MT
	II	SC	364	46.8	WT/MT
Mel 554	I	SC	0	45.3	WT/MT
	II	LN	118	44.9	WT/MT
Mel 592	I	LN	0	64.5	WT/MT
	II	LN	337	65	WT/MT
Mel 640	I	LN	0	6	*WT/WT*
	II	SC	366	7.7	*WT/WT*

Abbreviations: LN=lymph node; M=muscle; MT=mutant type; P=pancreas; SC=subcutaneous; WT=wild-type.

a Short-term cell cultures were established from initial (I) and subsequent metachronous metastatic lesions removed from cutaneous melanoma patients referred for surgery at the National Cancer Institute of Aviano (Italy). Short-term cell cultures were used in place of neoplastic tissues to get rid of contaminating normal cells that would have altered the measurement of tumor-specific BRAF^V600E^ frequency. To minimize modifications potentially arising with extended *in vitro* culturing, all cell cultures were utilized for molecular assays at the sixth *ex vivo* passage.

b Time frame of metastasis excision since the surgical removal of the first analyzed lesion.

c Percentage of BRAF^V600E^ mutant alleles was determined by pyrosequencing assay as described by Venesio *et al* (2008), with minor modifications.

d WT/WT, wild-type BRAF homozygote (% BRAF^V600E^<25); WT/MT, BRAF^V600E^ heterozygote (% BRAF^V600E^ 25–75); MT/MT, BRAF^V600E^ homozygote (% BRAF^V600E^ >75). The italicised entries help in differentiating WT/WT from the WT/MT heterozygous genotype.
